# Magic in the machine: a computational magician's assistant

**DOI:** 10.3389/fpsyg.2014.01283

**Published:** 2014-11-17

**Authors:** Howard Williams, Peter W. McOwan

**Affiliations:** Computer Vision, School of Electronic Engineering and Computer Science, Queen Mary University of LondonLondon, UK

**Keywords:** magic, optimization, AI, cards, jigsaw, computer, computational, creativity

## Abstract

A human magician blends science, psychology, and performance to create a magical effect. In this paper we explore what can be achieved when that human intelligence is replaced or assisted by machine intelligence. Magical effects are all in some form based on hidden mathematical, scientific, or psychological principles; often the parameters controlling these underpinning techniques are hard for a magician to blend to maximize the magical effect required. The complexity is often caused by interacting and often conflicting physical and psychological constraints that need to be optimally balanced. Normally this tuning is done by trial and error, combined with human intuitions. Here we focus on applying Artificial Intelligence methods to the creation and optimization of magic tricks exploiting mathematical principles. We use experimentally derived data about particular perceptual and cognitive features, combined with a model of the underlying mathematical process to provide a psychologically valid metric to allow optimization of magical impact. In the paper we introduce our optimization methodology and describe how it can be flexibly applied to a range of different types of mathematics based tricks. We also provide two case studies as exemplars of the methodology at work: a magical jigsaw, and a mind reading card trick effect. We evaluate each trick created through testing in laboratory and public performances, and further demonstrate the real world efficacy of our approach for professional performers through sales of the tricks in a reputable magic shop in London.

## 1. Introduction

A good magic trick is enjoyable for the audience; a great magic trick makes it seem, if only for a moment, that a miracle has occurred right in front of their eyes; Ortiz (1994) provides excellent discussions of what constitutes an exemplary trick. Magicians will go to great lengths to perfect a method that results in this type of theatrical impact. Taking into account all the constraints, both physical and psychological, that must be satisfied for a certain trick to exhibit magical qualities, performers will try to construct the best presentation possible. In this paper we refer to trick technology as being the combination of physical and psychological processes underpinning the technical effect. A trick's overall efficacy is dependant not only on the trick technology but also, and perhaps even more importantly, on the theatrical performance of the magician.

In this paper we focus on tricks that exploit mathematical techniques for their operation. The underlying mathematics behind magic tricks has a long and varied history; see Gardner (1956) and Diaconis and Graham (2012). Self-working tricks of these types, which rely on a hidden underpinning mathematical process rather than sleight of hand, can be powerful effects and are often included in card performer's repertoires to provide a break from the constant demands of manual dexterity. Usefully, mathematics based tricks give a clear set of constraints controlling the technical aspects of the trick. The card type and location in a pack can be indexed for example, building up a mathematical model of the physical effect which can be encoded and manipulated computationally.

Props and gimmicks can also provide a significant additional technical element. Props provide both theatrical window dressing and technical support in magic tricks; Christopher and Christopher (2006) describe many uses of such items. Often a prop's perceived role will be as an unassuming presence during performance, for example a simple table on stage, while its real role is fundamental to the method; Mayne (2005) shows how many such objects can be constructed and utilized. A gimmicked prop is one which resides in plain sight, for example a table, but performs some unseen role crucial to the trick's technical performance, for example a secret compartment in the table. Gimmicks that provide important trick technology may also be totally invisible to the audience. Hidden cue cards as memory aids are often deployed in card tricks, as shown in Aronson (1990), and the use of a human assistant who shares knowledge of the mathematical properties of a particular deck of cards underpins many powerful effects; see Kleber and Vakil (2002), Simonson and Holm (2002)and Lee (1950a).

The final element of trick technology is psychological. Human perceptual systems evolved to let us encode information from the surrounding environment. The processes by which this encoding occurs, and the way in which magicians manipulate and exploit these perceptual processes to create magical effects, has recently become an active area of scientific study, notably by Kuhn et al. (2008a). Magic tricks often rely on basic perceptual errors and illusions, many of which are documented by Robinson (1998), and the roles of misdirection and attention in magic have been extensively investigated in Kuhn et al. (2008b). Furthermore, the cognitive characteristics of playing cards such as favored audience choices, a staple of so many magic tricks, have long been of interest, initially to Fisher (1928) and latterly Olson et al. (2012). Related work in computer graphics examines the limitations of the human perceptual system, and how this can be exploited in various ways; see Harrison et al. (2004), O'Sullivan et al. (2003), and O'Sullivan and Dingliana (2001). Only through an understanding of the underpinning perceptual processes and the methods best suited to elicit the desired effect in performance, can magicians build convincing magical effects.

As is clear from the above, and from historical studies, there are multiple ways any one trick can be constructed and performed; Fitzkee (2009) provides a kind of lexicon of magical methods. Combining and recombining the trick technology elements in different ways can lead to different levels of magical impact, and computationally produces a combinatorial explosion in the space of possible solutions that can be difficult for humans to search; there are simply too many ways to put together variants of the trick-enabling elements to be able to try them all out to see which works the best.

Fortunately there are many computational techniques available to perform search and optimization in large data spaces; Russell and Norvig (2009) comprehensively deals with the subject. Genetic Algorithms (GAs), detailed in Goldberg (1989), and Simulated Annealing (SA), summarized in Russell and Norvig (2009), are used extensively in combinatorial problems. The idea of using computer systems as creative assistants, or even as creative entities, has been the subject of previous research, notably by Boden (1998), Bentley (2002), George et al. (1998), and Valstar et al. (2008) amongst many others. There has been some success in the use of Artificial Intelligence (AI) techniques to enhance computer gaming entertainment, by optimizing the mechanics of the games, see Liaw et al. (2013), and also the entertainment produced by the games as a whole, as with Yannakakis and Hallam (2007). To our knowledge, using AI methods to optimize magical effects in conjuring tricks remains a hitherto unexplored domain.

In the remainder of this paper we present a novel methodology for creating new magical effects and variants that relies on combining and optimizing both empirical perceptual and cognitive observations, and a mathematical model of the trick mechanics to generate novel trick technologies. The computer's role is that of a kind of digital magician's assistant that is able to find patterns and configurations that a human magician may struggle to identify. We demonstrate how this flexible approach can be applied to two different types of mathematics based tricks. Specifically we present a magical jigsaw puzzle designed by a GA that uses constraints derived from experiments on the vertical-horizontal illusion, detailed in Robinson (1998), and based upon the existing one dimensional geometric DeLand Paradox effect, documented by Gardner (1956). We also present a mind reading card effect based on cyclical De Bruijn sequences, described in Diaconis and Graham (2012), exploiting existing (Olson et al., 2012) and new empirical observations on the likeability of certain playing cards. Additionally this card trick relies on incorporating a mobile phone prop into the trick technology, which is used during presentation as both a memory aid and a method to reveal a card to the audience.

Finally, we show how we have evaluated the output of this approach to creating new tricks. We conducted experiments to measure the magical impact of the tricks in real life scenarios, and also produced the tricks as commercial products and placed them for sale in a well-known magic shop in London, UK. Sales of the products arguably form an in the wild validation for the methodology.

## 2. Materials and methods

### 2.1. Creating the magical

Our trick technology approach to creating new magical effects has three main framework components: a controlled problem domain determined by the type of trick the framework is working on (a formalization of all the elements, physical and psychological, that make up a trick, and a set of constraints placed upon these elements that make the trick viable and hopefully optimal), domain relevant perceptual and cognitive observations of psychological phenomena, and a computational search and optimization engine.

The problem domain needs to be identified and systematized, formalizing the parameters of the type of trick that the computational engine will work toward producing, in effect a mathematical model of the essence of the trick needs to be constructed. During this stage we exploited domain experts, magicians with performance experience, in order to fully understand how and why the type of trick under consideration works, to correctly abstract the various elements without missing crucial steps in the method. This technique of abstracting specialist knowledge to build a model is commonly used in various automated expert systems used for medical diagnosis and financial risk assessment, amongst others; see Russell and Norvig (2009).

The identification and analysis of the problem domain is naturally coupled with elements reflecting the psychological phenomenon being exploited during the trick, for example in the jigsaw trick we describe later we need to include a constraint on maximum line length increases commensurate with a spectator not noticing these length changes. Rather than model this phenomena directly we incorporate such constraints through encoding the results from subject empirical data, that is we run a series of experiments to qualify the effect and incorporate this data function in the overall model.

Finally, it is important to select a suitable search and optimization engine. The specific technique used is determined by the characteristics of the problem domain, and the type of data provided by the empirical investigations. Choosing a suitable technique is informed by previous applications of that technique that are similar in structure; Russell and Norvig (2009) provides many examples. Once the type of trick has been systematized, an effective technique can be identified and deployed.

**Figure 1:**
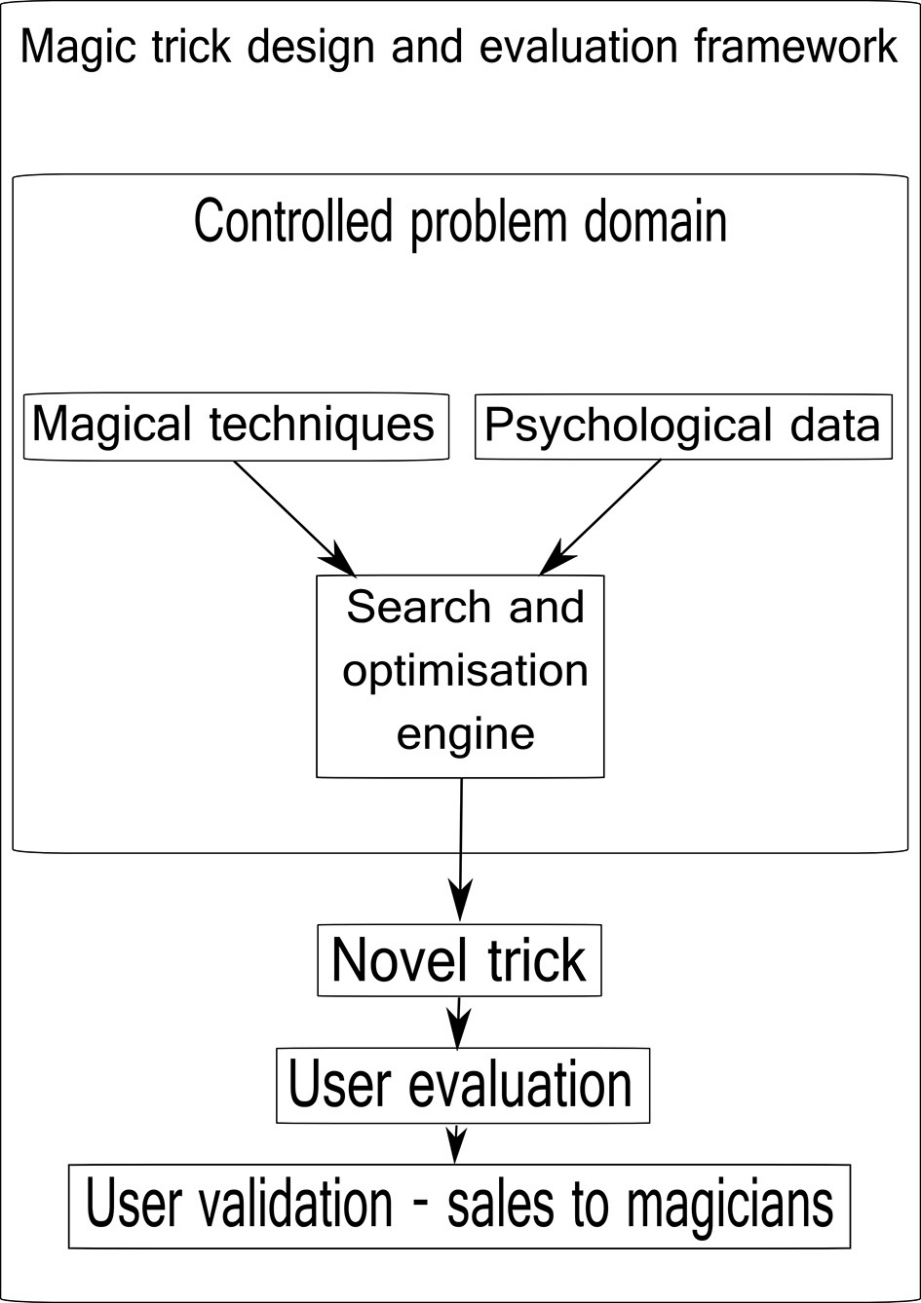
**The magic trick design and evaluation framework**. Known magical techniques are combined with psychological data relevant to the problem domain (a particular trick) by a computational engine that assists the design and optimization process. A new or optimized version of a trick is output. This trick is then evaluated in the field with performances for real people, and finally sold in a reputable magic shop in London, UK.

See Figure 1 for an overview of the framework components.

### 2.2. Evaluating the magical - externally iterating the optimization and evaluation loop

The computer model is configured to move toward an optimal goal as determined by the constraints of the mathematical model of the trick and the related constraints imposed by the psychological data. Optimization algorithms can find multiple potential solutions, these are referred to as local solutions, as there may exist one overall best global solution the technique does not identify. This issue around recovering a local or global solution is well known, and is dependent on initial conditions used, length of time the algorithm is run, and the algorithm tuning parameters used (see Russell and Norvig, 2009 for detailed discussions). In the case presented here the engine searches this space and the result delivered will be a working candidate for an optimized new trick. However, this working solution may not be the globally optimal solution and may, more importantly, not necessarily translate to a magical effect that performers can easily use. For example, the system may deliver a solution to a card trick that requires twelve cards to be dealt to a spectator, that they must then memorize and return to the magician! While being a solution that satisfies the model programming constraints, this is not a solution that would work in the real world.

Most of these issues are addressed by the psychological constraints imposed on the computer model, however to fully control for such non-practical solutions the outputs of the system need to be evaluated empirically with a real audience. We test the candidate tricks created by our systems by taking them out in to the real world and performing them for an audience. This audience is in essence a bank of experimental subjects who are unaware that what they experience has, in part, been designed by a machine. If necessary, the results from the empirical tests may feedback to the computational design phase, potentially informing the set of constraints used, though this step has not been necessary for the tricks explored in this work; non-computational factors, such as narrative and subtleties during presentation, are naturally refined during the evaluation phase. Once we have a final solution that maximizes the measured magical impact, and is also practical to perform, we undertake a final validation and evaluation of the results through productizing the trick and making it available for sale in a magic shop. This step provides clear evidence as to the viability of the created trick; it is assumed that a trick must reach some basic level of quality before a reputable shop will carry it as stock, and further that its purchase in exchange for money indicates, in a very direct way, the success or otherwise of a product with our specific target user base (magicians).

### 2.3. Measuring magical impact

To test the candidate and final versions of tricks we use an evaluation questionnaire that participants can be asked to complete after witnessing a trick. The intention is to measure their overall experience of the trick—some people dislike magic tricks, even if they are somewhat surprised or amazed by what they have seen. Equally, a participant may know or guess the fundamental techniques at work in a given trick, and therefore not find it to be an especially magical experience, but may still enjoy the particular presentation offered.

We use two scales to capture how much, in general, participants enjoy magic tricks, and also, separately, their enjoyment of the particular trick they have witnessed, we use: an ascending enjoyment scale of 0–4, mapped to the phrases: “Hate(d) them(it),” “Dislike(d) them(it),” “Neutral,” “Like(d) them(it),” “Love(d) them(it).” Data gathered about whether participants enjoy magic tricks in general can be used to view the rating of a particular trick in a different light. Someone who genuinely does not like magic tricks is much less likely to enjoy a particular trick and vice versa. It is likely that when asked about how much they enjoy magic in general, participants would likely recall the best experiences they have had of magic, rather than some average they calculate. Thus, if adjusting the rating scores for a particular trick according to a participant's general rating of magic, it is to be expected that the average score for a trick would drop, but may provide a better overall measure. A calibrated rating can be calculated using the formula: *CalibratedRating* = *TrickRating* + (*TrickRating* − *GeneralRating*). This way, if, for example, a participant dislikes magic in general, but loves a particular trick, the calibrated rating will positively reflect this. This method accentuates weak ratings. A useful measure of how well a trick is received by a group of participants is the difference between the average (mean) rating given to magic in general, and the average (mean) rating given to the particular trick. The smaller the value the better (the theoretical minimum is minus four, though anything close to zero is very good).

**Figure 2:**
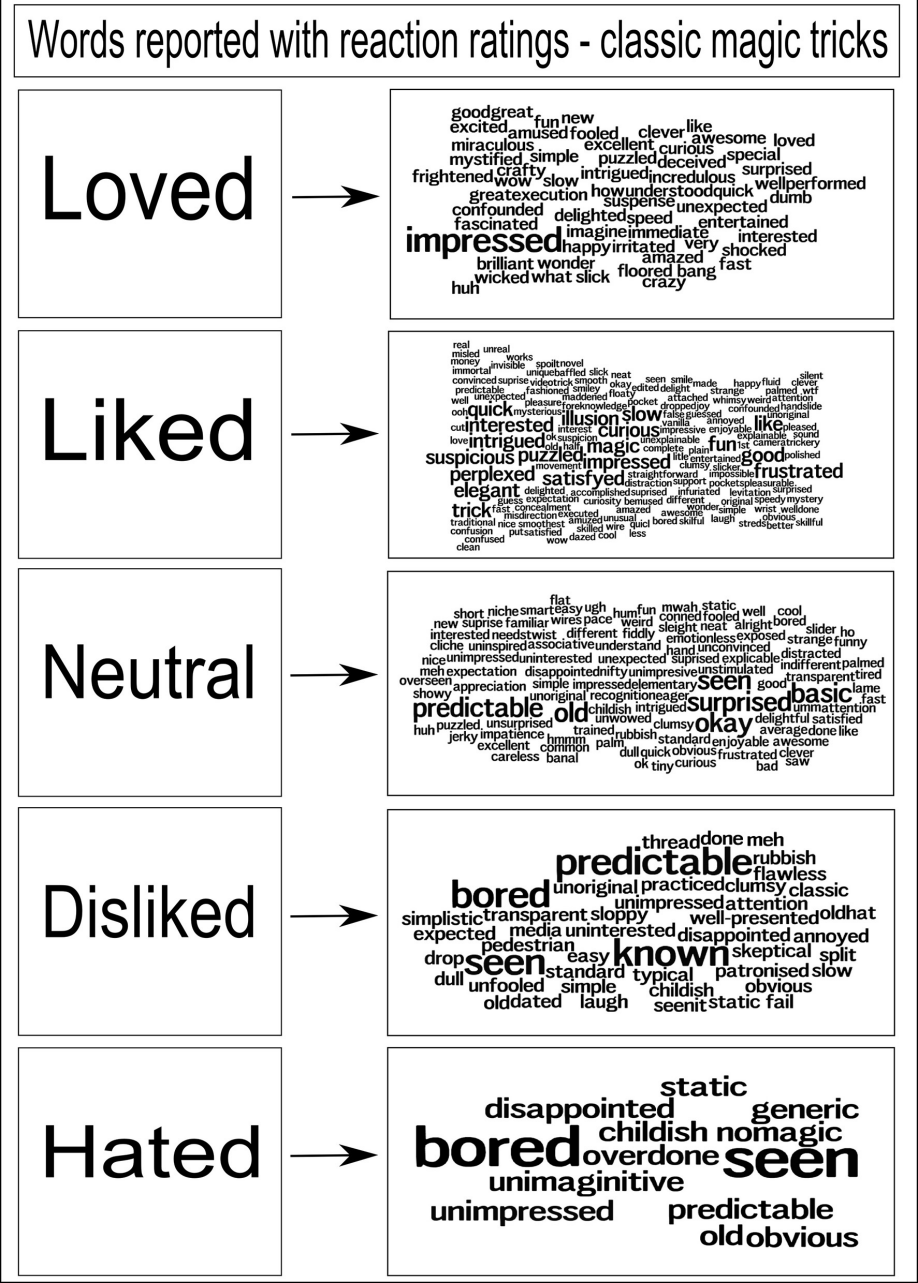
**Word clouds representing gathered responses from people shown classic magic tricks**. The larger the word, the more often it was reported as a response to a trick. We recorded a participant's rating of a trick, along with their word reactions; each cloud of words shows the words that were recorded for each rating. During development of the evaluation framework, this list was distilled to a core set of words to use. N.B. Initial evaluations, as shown in the section discussing the magical jigsaw puzzle, allowed a greater range of words to be selected.

We performed experiments (*N* = 96) asking participants to freely choose words to describe their reactions to a range of classic magic tricks, the results of which are shown in Figure 2. The intention here was to gather data about the type of descriptive words people use when asked to give a reaction to a magic trick. The participants were recruited from university mailing lists, and from disseminating details of the experiment on Twitter. To simplify the questionnaire, we did not ask for age, gender or country of origin data from the participants. From these words, we observed those most commonly used, and made a selection available on our questionnaire, covering a spectrum of emotions, as choices for participants in our later evaluations of the generated tricks. The distilled list of words participants are asked to select from to represent their reaction to a trick is: Bored, Surprised, Obvious, Neutral, Impressed, Predictable, Amazed.

The holistic summation of the experience provided by these emotional spectrum words provides an additional, qualitative, view of the experience of a trick for a spectator, a measure deliberately separate to the enjoyment rating. We have intentionally not numerically quantified these words. However, more usefully, the words provide additional evidence to the trick designer as to how the trick is received. The quantitative measure of enjoyment provides a way for participants to score the trick numerically, while selecting words allows a spectator to disambiguate that perhaps they enjoyed the trick (high enjoyment rating) but found it predictable. It is arguable that a professional performer would only be satisfied if a trick generated something akin to an “Amazed” response, regardless of the enjoyment rating. It is equally arguable that the rating, how much an audience enjoyed the experience, is the key factor. The intention is to try to understand the way that the tricks are experienced, in a more comprehensive fashion than simply the numerical score of enjoyment.

To further help identify weak points in the trick, subjects were also asked to write freely about any moments when they felt something suspicious might have happened, and about how they thought the trick works.

Collecting this kind of data provides a numerical indication of how much a trick has been enjoyed, and also some more qualitative data about the subjective experience of a generated trick. These observations can be compared to similar data collected from people that have been shown traditional, known to be effective, magic tricks.

Arriving at a measure of what is experienced phenomenologically by someone witnessing a trick is difficult; our approach provides a useful, practical view of a trick's magical and entertainment impact, without the complexity of deeper philosophical questions about the nature of magical experiences.

In the following sections we describe two magical effects, designed using our conceptual framework: a magical jigsaw puzzle, and a mind reading card effect. Table 1 shows a summary, for reference, for each trick, of the three components necessary to create the trick.

**Table 1:** **Summary of psychological data, constraints, and AI technique applied to design each trick**.

**Trick**	**Psychological observations**	**Constraints**	**AI technique**
Jigsaw	1. Threshold of length increase detection for rectangles.	1. Physical constraints on jigsaw pieces that make up two viable puzzles.	1. Genetic Algorithm.
	2. Number of jigsaw pieces that can be practically assembled.	2. Optimal targets for each of the three psychological components, with upper bounds outside of which solutions are unacceptable: (a) Length increase. (b) Number of jigsaw pieces. (c) Number of rectangles.	2. Rectangle packer (to generate tilings).
	3. Number of rectangles easily countable.		
Card trick	1. Likeable cards.	1. Cyclical sequence of cards defined by user specified categories.	1. Simulated Annealing procedure.
	2. Cognitive visibility of mobile phone gimmick prop.	2. Min/max depth of generated tree.	
		3. Positioning of special (e.g., Liked) cards.	

### 2.4. A magical jigsaw

We applied our framework to the problem of making an optimally magical jigsaw puzzle, where printed graphics elements appear and disappear depending on how the same jigsaw is constructed. This jigsaw is based on The Principle of Concealed Distribution, an old technique, first developed seriously in Gardner (1956): the geometrical redistribution of segments of one shape among a number of other shapes such that the magnitude of increase in the area of the remaining shapes is imperceptibly small. The DeLand paradox is an early example of this type of effect, documented by Gardner (1956). An image showing objects is rearranged such that one of the objects appears to vanish, but in fact has been incorporated into an increase in length of the remaining objects. These types of effect were very popular in the late 1800's and early 1900's; Sam Loyd's Get Off The Earth from 1896 followed The Magic Egg by Wemple & Company, from 1880. DeLand's version appeared in 1907.

Converting the one dimensional DeLand paradox to a two dimensional jigsaw allows for greater flexibility in how the shapes can be positioned and redistributed, while simultaneously increasing the sense that something physically impossible has happened; it is typical to assume a jigsaw puzzle can be put together in only one way.

Previous versions of this type of effect have the rectangles displayed vertically in both configurations of the image. We noted the vertical-horizontal illusion reported in Robinson (1998): a line displayed vertically will appear longer than an identically sized line displayed horizontally. A jigsaw puzzle operates in two dimensions, and allows rotations as well as translations of pieces giving the opportunity to usefully exploit this perceptual illusion. We conducted psychophysical experiments to determine the upper limit of rectangle length increase that could be applied before subjects would notice the difference—we investigated the effect on length perception of showing multiple rectangles vertically, followed by multiple rectangles displayed horizontally, and a mixture of the two orientations.

We also investigated the effect of using increasing numbers of rectangles and how this would affect the participant's experience. If too many rectangles are shown they become difficult to count accurately in a reasonable time; the impact of the effect would be diminished as the spectator would be too engaged in counting. Conversely, more rectangles on display can improve the effect, as it is harder for a spectator to determine the method by mentally recombining rectangles. As the trick relies on the subject knowing there are different numbers of rectangles in the two different jigsaw configurations, we conducted experiments to determine the number of displayed rectangles that could be easily counted without error in a reasonable time.

A jigsaw may be made up of different numbers of pieces, of different basic shapes (rectangles and squares). These must all fit together seamlessly with connecting lugs and gaps for each piece, in both configurations. Crucially, a performer needs to be able to construct and then reconstruct the puzzle efficiently, without mistakes. However, more pieces make the method behind the effect harder to resolve in a spectator's mind. We conducted experiments to determine how many pieces could be reliably constructed in a reasonable time.

These factors determine what makes a good jigsaw trick for both the performer and the spectator. There are other issues of a more basic geometrical nature for a jigsaw designer to contend with, such as what shapes of pieces to use, where to place them, and where to position the lugs and gaps on each piece to make viable puzzles. Further, where each rectangle must be positioned so that after rearrangement the desired decrease in the number of rectangles is achieved.

For a human designer, this leads to an intractable combinatorial explosion of possibilities for jigsaw designs. However, GAs are excellent optimizers for such challenges, as shown in Goldberg (1989). GAs are able to perform searches through large, complex problem spaces that contain (undesirable) local optima. The jigsaw is in fact a multi-objective optimization problem; conflicting constraints mean there is not necessarily a single solution where each objective is optimal; a balance may need to be struck.

We used data from our psychophysical experiments as objectives in the GA's fitness function. A range of values for each of the constraints will result in workable, though not optimal, solutions. Other parameters affect the viability of each candidate solution during the design process; for example, a basic requirement is that the pieces of the jigsaw must fit together to form the same basic overall shape, covering the same surface area (i.e., no gaps).

The model, encoded as a binary bit string by the GA, that represents each candidate jigsaw solution consists of:

1. Basic overall shape and size of jigsaw (e.g., NxN square).
2. Number of jigsaw pieces.
3. Shape and size of each piece.
4. Configuration of lugs and gaps on each edge of each piece.
5. Number of whole rectangles on the first jigsaw configuration.
6. Size of rectangles.
7. Co-ordinate positions and orientations of pieces in each of the two jigsaw configurations.
8. Co-ordinate positions and orientations of rectangles on the initial jigsaw.

A discretized co-ordinate system was used for all sizes, positions, and orientations.

The specific constraints used in fitness evaluation are detailed below. Hard constraints (denoted [HARD]) are those that define a viable jigsaw (i.e., a candidate solution that does not meet the hard constraints is not a valid solution; e.g., there may be lugs that do not have a gap to slot into). Optimization constraints (denoted [OPTI]) are those to be minimized or maximized to search for the best, as defined, magic jigsaw:

1. [HARD] Area of first and second jigsaw solution covered by generated pieces. This should cover the same area as the defined shape of the desired solutions, with no gaps.
2. [HARD] Number of pieces that are fully connected by jigsaw lugs in the first and second jigsaw solution. All lugs must connect to a gap. No spare gaps.
3. [OPTI] Number of whole rectangles of the required size on the second jigsaw. Minimize this number (this defines how many rectangles have “vanished”).
4. [OPTI] Number of rectangle fragments on the second jigsaw. Minimize this (zero is optimal).
5. [OPTI] Spatial distance of rectangles from configurable points on the jigsaws. Pleasing designs cover the surface of the puzzle more evenly (relevant to the spectator).
6. [OPTI] Total number of jigsaw pieces, scored from a scale mapped from experimental data (relevant to the performer and the spectator). Eight pieces is defined as optimal. Minimize the deviation from this.
7. [OPTI] Total number of rectangles, scored from a scale mapped from experimental data (relevant to the spectator). Minimize this.
8. [OPTI] Rectangle orientation score for each jigsaw, scored from a scale mapped from experimental data (relevant to the spectator). Optimally all rectangles on the first solution are vertical, while all on the second are horizontal.

This type of multi-objective problem needs a specialist GA algorithm; we used a NSGA-II (Deb et al., 2002) derived GA coupled with a rectangle packing algorithm (Lodi et al., 2002). Rectangle packers are used to efficiently pack shapes into containers. We applied the standard NSGA-II algorithm with the constraints outlined above, using the rectangle packer to generate valid candidate puzzles from a given set of basic shapes. The algorithm converges to solutions in less than fifty generations of the GA's iterative process—the number of pieces and number of rectangles increases the complexity. The computation time to design the example featured was approximately 2 min on a desktop PC with an Intel Core i5 processor.

**Figure 3:**
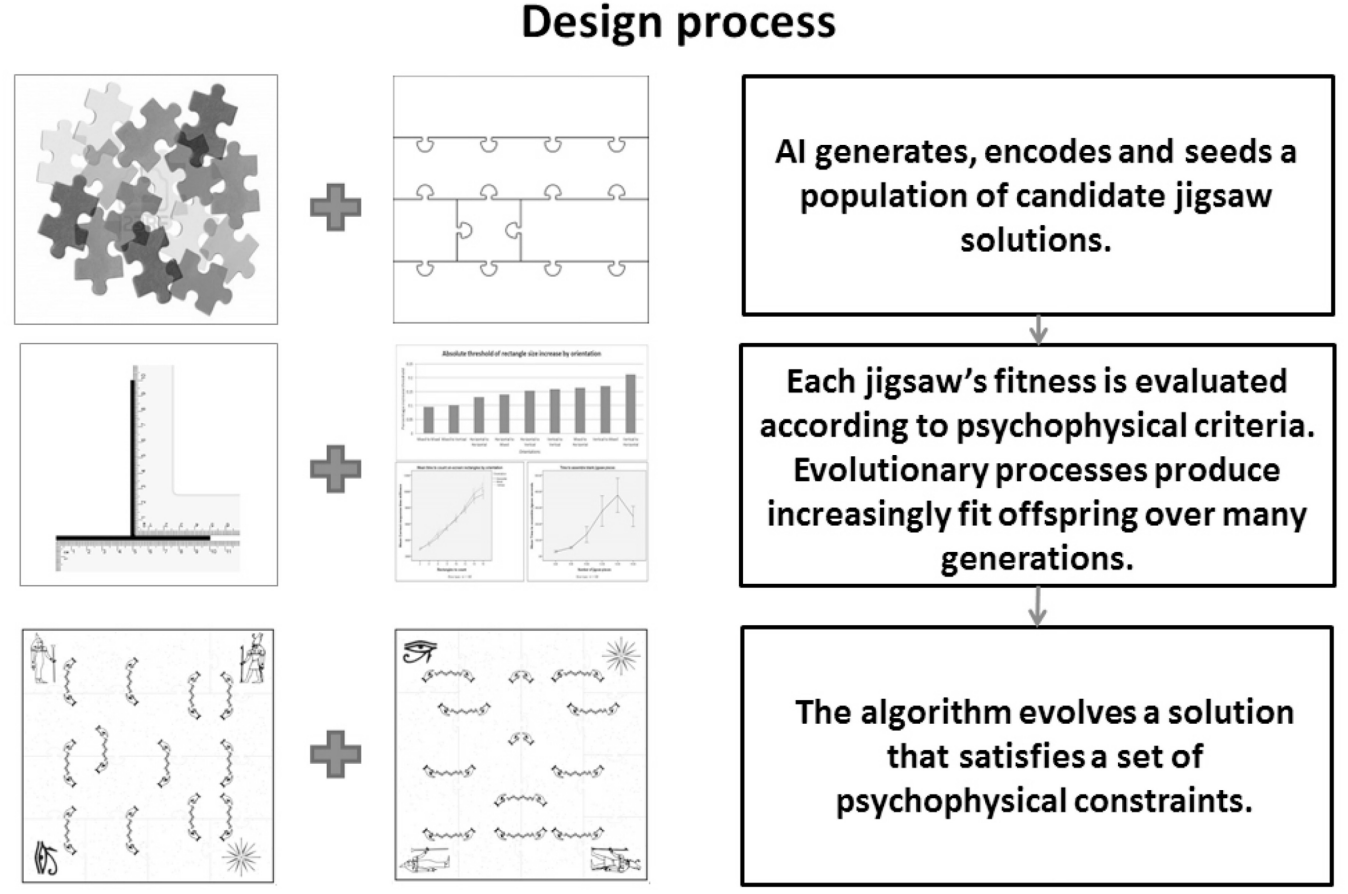
**The Genetic Algorithm driven jigsaw design process**. Geometric and empirically derived psychological constraints are used by a GA to design a perceptually compelling jigsaw magic trick.

See Figure 3 for an overview of how the framework was applied to the jigsaw design problem.

With this optimization configuration our automated system is capable of synthesizing the various geometric and perceptual elements we have discussed to design novel jigsaw tricks to flexible specifications.

### 2.5. Jigsaw results

**Figure 4:**
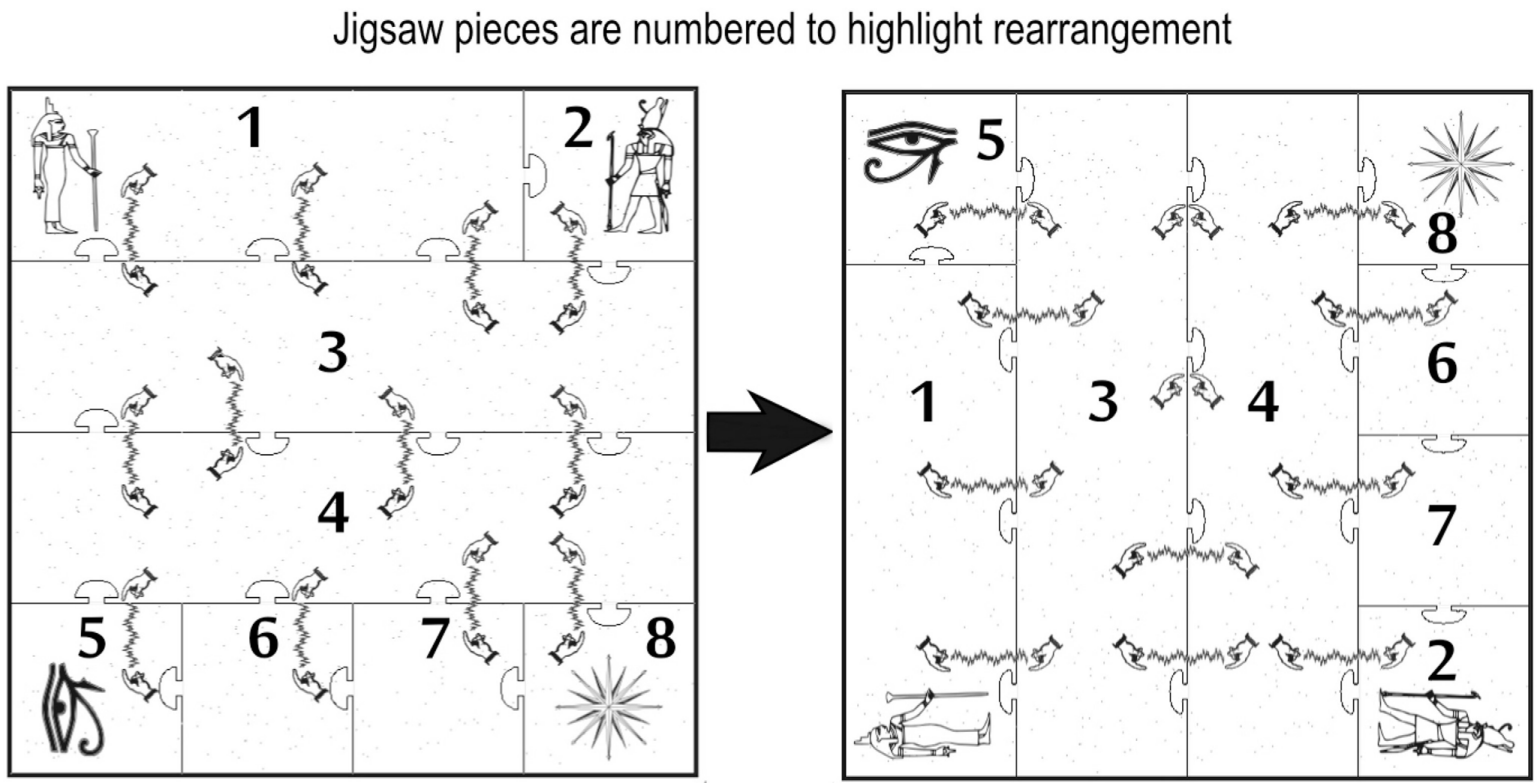
**The magic jigsaw**. The first configuration, shown on the left, depicts 12 “spells,” two of which subsequently seem to vanish in the second configuration, shown on the right. Each “spell” in the second configuration has grown imperceptibly in length. The numbers on the pieces have been added here to help show where each piece starts and ends in each configuration; the real jigsaw as sold is not numbered.

By way of illustration we have chosen one of many outputs possible from the jigsaw design system. The jigsaw created by the system is an eight piece interlocking puzzle showing twelve rectangles on its surface; after rearranging the pieces the surface displays only ten rectangles. Here we show a design themed around Egyptian mythology, where the rectangles have become “spells” cast between pairs of hands. See Figure 4. During the puzzle's reconstruction, the remaining rectangles are larger than those in the original image but an observer should not notice this length increase.

Using the method of constant stimuli, described by Laming and Laming (1992), we determined the absolute threshold of the amount of change in the length of rectangles able to be perceived. This threshold is defined as the amount of change in length that participants are able to accurately report for more than 50% of stimuli.

Participants were shown pairs of sequentially presented images, separated by a blank screen. Each pair consisted of an image of six rectangles of either all vertical, all horizontal or mixed orientations, shown for one and a half seconds, followed by a blank screen for 1 s, followed by a second image of six rectangles also of either all vertical, all horizontal or mixed orientations. For each image, all rectangles were randomly positioned on screen with none overlapping. The group of rectangles in the second image would either all be the same length as all those in the first image, or would all increase by a certain percentage. The increase ranged from 0 to 30%, in 5% increments. A pair depicting a certain percentage length increase was shown to the participant ten times; the pairings were displayed with a random order of presentation. The participants were asked only to determine if the lengths of the second set of rectangles had increased in comparison with the rectangles in the first image; a yes or no. The threshold is derived from regression fitting a line to the detection of increase data.

As anticipated, the vertical-horizontal illusion is evident; the largest absolute threshold value of 21.1% size increase was in effect when subjects were shown an image containing all vertical rectangles, followed by an image containing all horizontal rectangles (denoted VH). The complete set of combinations of orientation resulted in the following absolute thresholds (H = Horizontal, V = Vertical, M = Mixed): VH (21.1%), VM (17.0%), MH (16.3%), VV (15.8%), HV (15.3%), HM (14.0%), HH (13.0%), MV (10.1%), MM (9.5%).

These results on length increase echo recent findings from Harrison et al. (2004) on perceptible size increase in the links in an animated articulated figure when attention is not fully focussed on the relevant links; in this scenario they also report that size increases of over 20% can go unnoticed. This may point to a general psychological effect: that higher thresholds of size change perception may be present where attention is not fully focussed.

**Figure 5:**
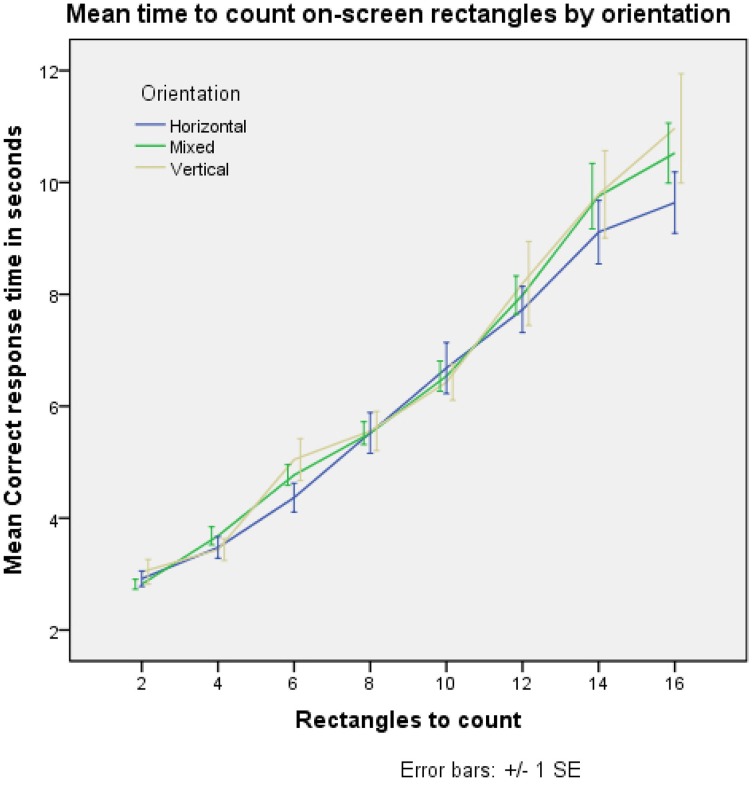
**Increasing the number of rectangles on screen for a participant to count linearly increases the time taken to accurately count them**.

The observer of the trick is required to count the number of rectangles on the puzzle; we investigated the amount of cognitive load this produced. Previous studies, see Mandler and Shebo (1982), suggest a response time of 250–350 ms per item counted above the subitizing range (the number of items that are able to be counted in a negligible amount of time without much cognitive effort; generally thought to be up to 4 items). We performed our own online experiment to determine the rate at which subjects (*N* = 49) were able to count rectangles on a screen, see Figure 5. During our experiment, it was necessary for the participants to find and press an on-screen button, indicating the numbers of rectangles they had counted, and another button to submit their count. From the data, it is estimated that this process takes approximately 2800 ms. Adjusting our data for this, and calculating a per item response time, it appears that as the number of rectangles increase, the underlying time increase per rectangle also increases slightly; this may be explained by participants being more likely to lose count while viewing more rectangles, and therefore having to restart. Further, for larger numbers, any time taken by a participant to check the count is likely higher. Times were recorded only for correct counts. From our data, counting the rectangles takes between approximately 160 ms per rectangle (for 4 rectangles) to approximately 470 ms per rectangle (for 16 rectangles).

**Figure 6:**
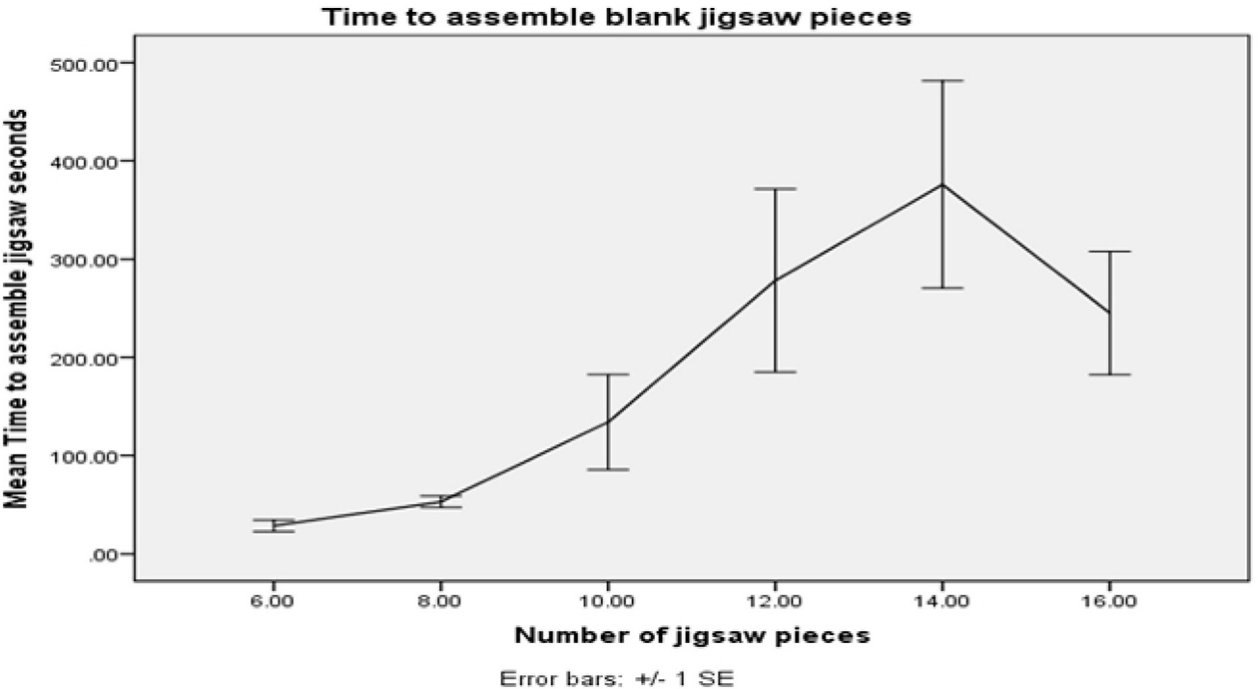
**Increasing the number of pieces of a blank jigsaw to assemble as a task seems to become non-trivial for the participants for jigsaws with greater than 8 pieces**.

A trick with too many pieces may take the performer too long to assemble, and be prone to error. After a trial study (*N* = 5), it appears that the time taken for subjects to assemble blank jigsaw pieces into a square shape becomes highly variable beyond eight pieces. See Figure 6. This gives us another constraint we include in the optimization.

We empirically evaluated the magical effect of the jigsaw (*N* = 100) and compared the ratings from those gathered for the classic magic tricks (*N* = 96). Unfortunately, the idea to record participant's general ratings of magic came only after the classic magic trick experiment had been run, therefore it is only possible to report unadjusted ratings for these tricks (i.e., the ratings are not calibrated by a participant's rating of magic in general).

The participants for the trick evaluations were recruited from university mailing lists, and from disseminating details of the experiment on twitter. To simplify the questionnaire, we did not ask for age, gender or country of origin data from the participants. We showed participants videos of each trick, and asked them to rate their enjoyment of the trick on the scale [Hated (=0) through Loved (=4)]; for the jigsaw trick experiment we also asked the participant how much they enjoyed magic generally, using the same scale.

Different versions of the jigsaw trick were produced, to investigate the effect of narrative. The jigsaw trick videos shown were: (1) The full jigsaw trick, with a narrative describing the events shown, which frames the trick in a mythological story based in ancient Egypt; the vanishing rectangles are “spells.” (2) The same trick, but with no narrative describing the events shown; the jigsaw is simply rearranged on screen in a mechanical way, with a finger pointing to the “spells.” (3) The jigsaw is rearranged on screen, but no “spells” vanish, therefore nothing magical has occurred; a narrative is supplied, very similar to the Egyptian themed mythological story supplied previously, but with a different ending that does not reference anything vanishing. (4) The jigsaw is rearranged on screen, but no “spells” vanish, therefore nothing magical has occurred; no narrative is supplied.

The classic tricks shown were: (1) A skilled magician showing a cup vanishing, just before being smashed; no sounds or patter. (2) A skilled magician showing a piece of cloth vanishing; no sounds or patter. (3) A skilled magician showing a piece of paper floating in the air; no sounds or patter. (4) A skilled magician showing a cigarette being broken in two, then magically repaired; no sounds or patter. (5) A skilled magician showing a giant coin suddenly appearing; no sounds or patter.

**Figure 7:**
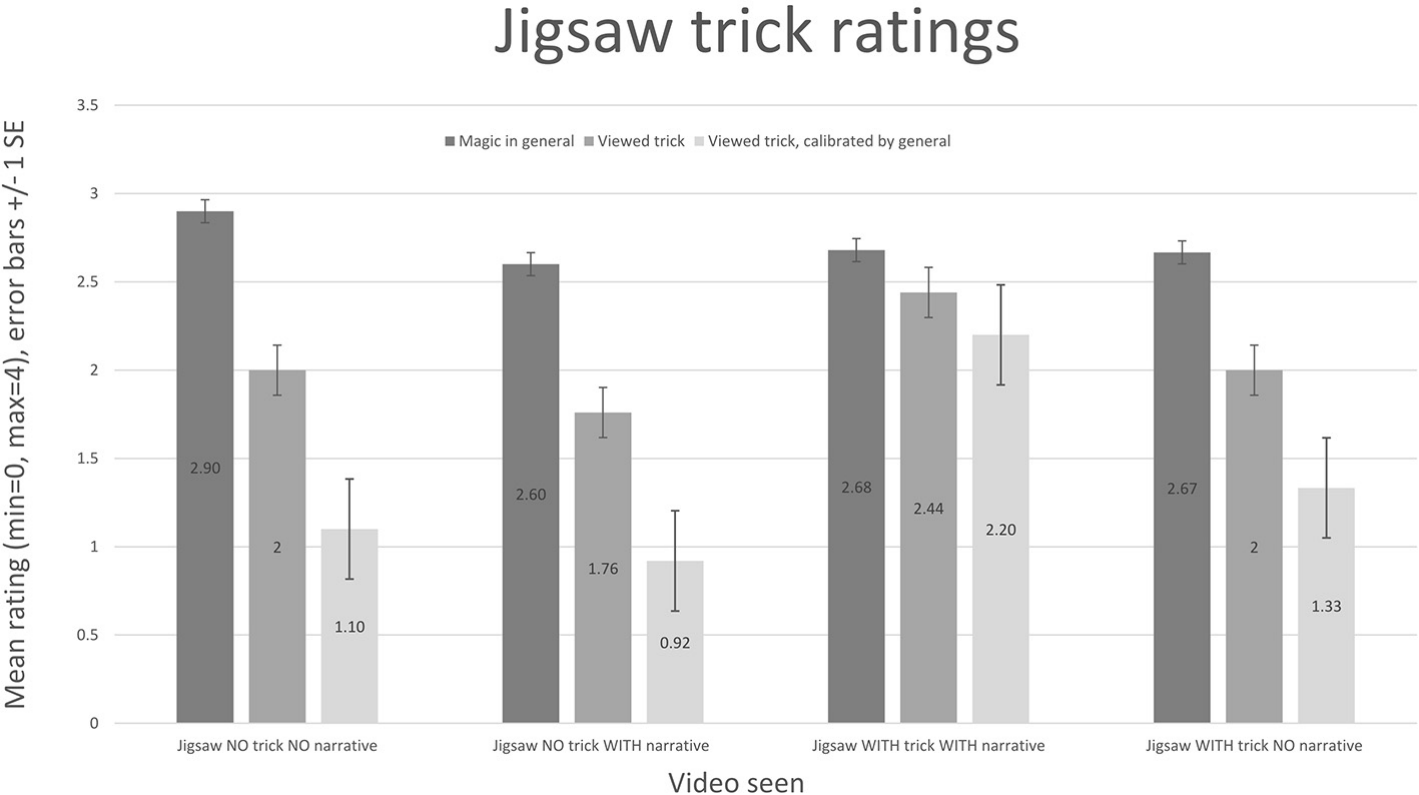
**The jigsaw enjoyment ratings are shown, along with the reported enjoyment of magic in general by the viewers of each video**. The third rating is a calibrated value, based on the formula *CalibratedRating* = *TrickRating* + (*TrickRating* − *GeneralRating*). The jigsaw trick with a full narrative scores comparably with classic tricks. The calibrated values emphasize weak ratings. The difference between the general rating and the trick rating, for the full jigsaw trick, is 0.24.

**Figure 8:**
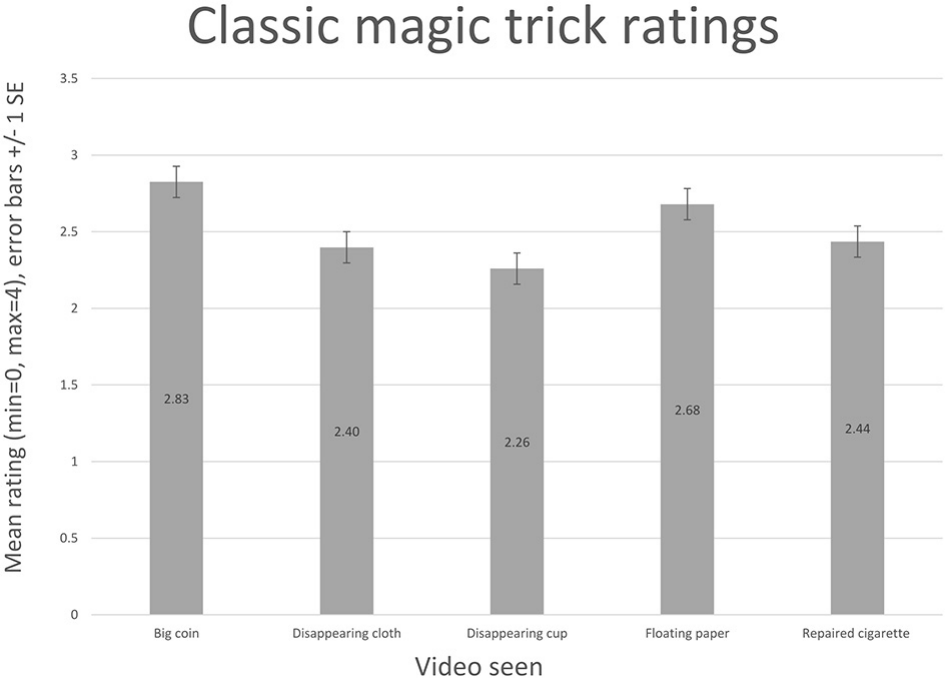
**The enjoyment ratings for classic magic tricks are shown**.

For ratings of each trick, see Figures 7, 8. The jigsaw trick with a full narrative scores comparably with classic tricks (though they are presented without a narrative). The calibrated values emphasize weak ratings. The difference between the general rating and the trick rating, for the full jigsaw trick with a narrative, is 0.24. The difference between the other video ratings and their associated general ratings is much higher: jigsaw, no trick, no narrative (0.9); jigsaw, no trick, with narrative (0.84); jigsaw, with trick, no narrative (0.67).

It is interesting to note the role that introducing a narrative to the jigsaw trick has on its enjoyment rating; the worst score comes from the version where nothing magical occurs, and no narrative is supplied (unsurprisingly). Introducing a narrative to this version improves the enjoyment of the experience; however, the version showing a magical effect, but with no attached narrative, scores better (using the difference metric). The implication is that if the viewer is expecting a magic trick and nothing magical happens, this has a detrimental impact on their enjoyment, even if a story is told. Narrative, however, does play a large role: the highest scoring video supplies both a narrative and a magical effect. While it might be expected that the version that shows a magical effect but has no narrative would score similarly to the classic effects (also presented without narrative), it should be noted that the jigsaw trick arguably relies more heavily on the narrative to explain what is occurring than the other tricks—crucially to highlight that something has vanished—the classic effects are all easy to understand without an accompanying narrative.

Participants who viewed the jigsaw tricks were also asked to select a word to describe their reaction to the tricks they had witnessed. This evaluation was performed with a longer list of words than the distilled list we use for our, later developed, standard evaluation; the longer list was selected from words describing the classic magic tricks. Not all participants (from *N* = 100) chose to select a word to describe their reaction. What follows is a breakdown of the number of times a word was reported by a participant after viewing the full jigsaw trick (with vanishing “spells” and a narrative). Most responses are positive, or express a sense of something unexplainable having occurred: Bored (1), Clever (5), Clumsy (1), Confused (3), Cool (4), Disappointed (2), Dull (5), Easy (1), How? (6), Interested (5), Predictable (2), Puzzled (5), Rubbish (1), Skeptical (3), Simple (4), Slick (2), Surprised (1), Unexpected (2), Wonder (1).

In a final qualitative study (*N* = 7), when asked to describe how the trick worked, or any suspicious moments arising, four participants reported having no idea how the trick worked, two made accurate guesses but were hesitant, while the remaining participant explained the trick as an optical illusion.

A physical version of the jigsaw was productized as a wooden puzzle, laser cut and printed, and packaged with instructions for sale. The jigsaw was included as part of the inventory in a reputable and well established magic shop in London, and the two runs of the product sold out (30 units). The cost for the jigsaw was set in conjunction with the shop owner, an experienced salesman of magic tricks, who was able to provide what, in his professional opinion was a competitive price compared to other similar tricks. This is direct evidence of the efficacy of the methods presented in this paper to create novel, practical, and saleable magic effects. These sales are considered as evaluation metrics in a research project rather than as a commercial product, but it is worth noting the shop requested further stocks.

### 2.6. Combinatorial cards

We then applied our framework to the creation of a mind reading card trick. Using the conceptual framework outlined, we created a flexible automated system capable of searching for user specified combinatorial structures in decks of regular playing cards that can be used for magic tricks, taking into account cards that would be most likely selected by an observer.

The use by magicians of cyclical combinatorial structures in mind reading effects, for example De Bruijn sequences—cyclical sequences of objects in which each unique subsequence of a given length appears once—have been extensively investigated by Chung et al. (1992) and Diaconis and Graham (2012). There are well known computational algorithms capable of generating particular types of sequences, detailed in Knuth (1997), Fredricksen (1982) and Stein (1961); here we build on these to devise an algorithm able to produce cyclically ordered decks of cards to flexible specifications, for use in magic tricks.

Finding cyclical structures can be a difficult task for a human trick designer: the number of permutations of a deck of 52 standard playing cards is a huge 52 factorial (8 × 10^67^). A cyclic sequence of cards is of benefit to a magician during performance, as cutting a deck of cards allows a false sense that the cards have been shuffled (see Hugard and Braue, 1974 for extensive discussion of card shuffling techniques), without disrupting the cyclical sequence.

The cognitive characteristics of playing cards have been previously studied by Fisher (1928). Recent work by Olson et al. (2012) shows that certain cards tend to be liked in preference to others. For example, the picture cards (Jack, Queen, King) and Aces are preferred, along with the Heart and Spade suits.

To encode the card characteristics in a form suitable for our framework we allocated individual playing cards as belonging to a number of categories depending on their features—for example the King of Hearts belongs to the categories: Heart, Red, Picture Card, High Value. We define the Liked (and Not Liked) category by using the Likeability index, an ordered ranking of how well liked each playing card in a standard deck is when compared to other cards, described by Olson et al. (2012).

In many mind reading effects involving playing cards a magician will dispense cards from a pre-ordered deck and subsequently ask a number of vague innocuous sounding questions to covertly recover the information needed to reveal the card identity, for example: “are you thinking of a red card?”. This process is referred to by magicians as fishing (discussed in detail in Aronson, 1990), magically arriving at a specific, supposedly secret, card while not making it look like they are asking too specific a set of questions. To elicit a magical effect the questions must be perceived as vague and almost inconsequential. The varied approaches to the bank of fishing questions often differentiate the quality and impact of these effects. A classic example is Larson and Wright's Suitability, described in Diaconis and Graham (2012): a 52 card deck is ordered in such a way that dealing three consecutive cards from any position in the deck yields a unique set of three Suits. Other orderings can be found such that consecutive cards may be differentiated by multiple categories; for example, Suits, Color, and Picture Cards. A suitable set of fishing questions then need to be deployed to recover the actual identity.

These kinds of orderings of cards characteristics may be represented as a computational tree structure, defined in Knuth (1997), a category at each level determining which tuples (sequences) of cards are placed at which node (branching points), ending in leaf nodes that contain only one tuple of cards of the requisite length. The trick Suitability's tree has only one level beyond the root (the start node), thus requiring only one fishing question per card (which suit it belongs to).

Generally, the shorter the fishing trip of questions is, the more magical the effect. Simon Aronson's trick Simon-Eyes, described in Aronson (1990), can also be analyzed as a tree structure; Simon-Eyes' tree has multiple levels. The pay off is that only two cards need be dispensed, and the questions are never met with two negative responses—for example, if the route through the tree leads to an enquiry suggesting one of the cards is low valued, then at least one of the two cards will be low valued. This is a powerful technique for a magician to deploy, as it builds confidence for the observer that the magician is performing something other than simple question and answer sessions.

**Figure 9:**
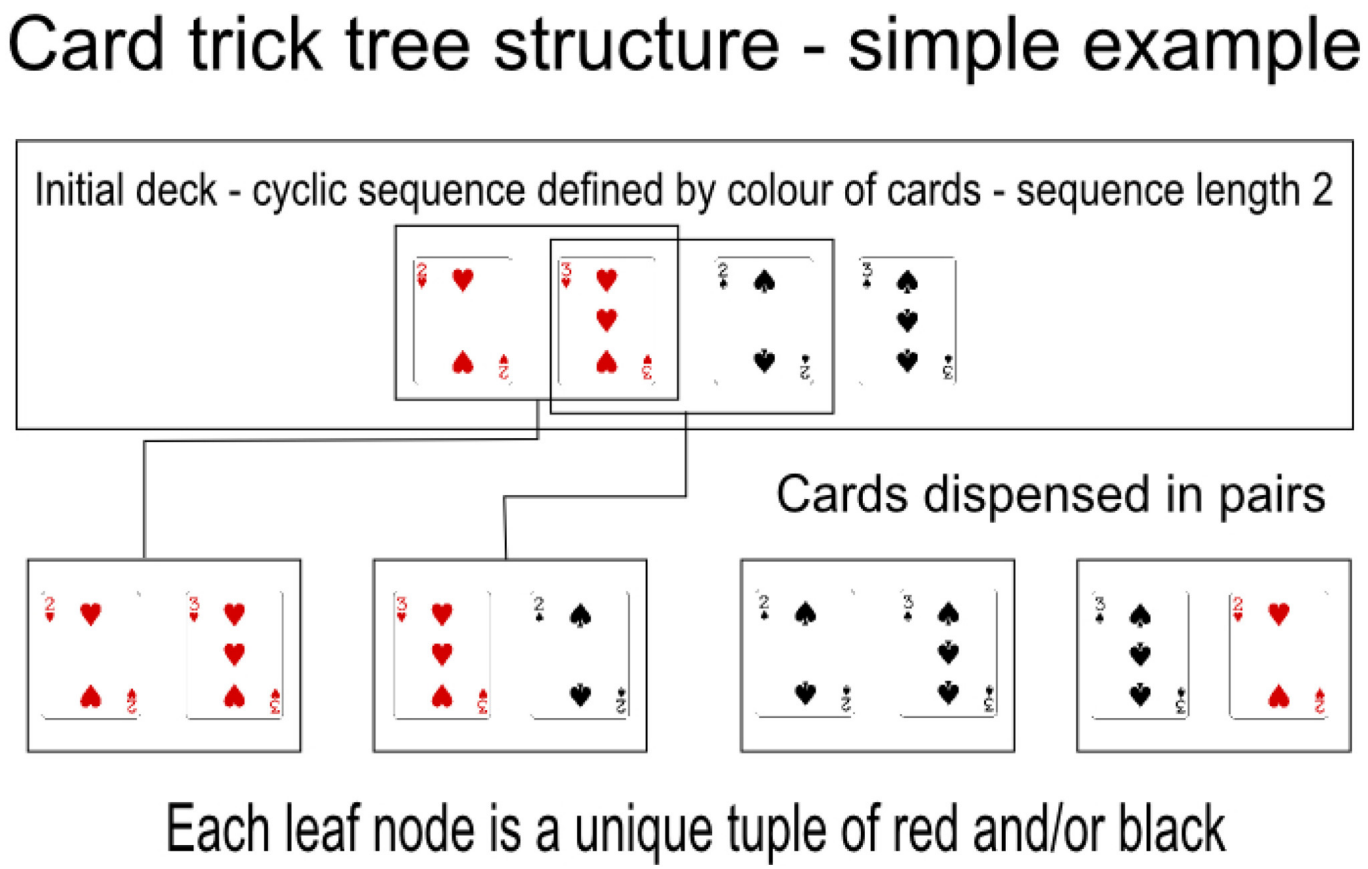
**A simple example of how a deck can be cyclically ordered, breaking down into tuples of length two, within a four card deck**. This is a very simple tree with only one level below the root. Each pair of cards dealt from the deck will be a unique sequence of red and black cards. A magician can dispense two consecutive cards from the deck to a spectator, for example the 3♡ and the 2♠, then fish from the spectator the color of each card (in this case Red then Black). Finally, the magician can ask the spectator to select one of the cards. If the spectator selects the first card, the magician knows that it must be the 3♡.

In the context of our framework we wish to encode a tree based structure representing a cyclically ordered set of playing cards that deconstructs at each level of the tree into a set of cards distinguished by category. Additionally at each leaf node there must be only one set of cards of a given length and all cards in the deck must be in at least one leaf node. See Figure 9 for a simple example of this type of structure as used in a magic trick.

Different orderings of cards result in different tree structures of variable quality, depending on their maximum and average depths (related directly to the number of questions required to traverse from the root to a leaf node). The magical potential of an ordering that also relies on the Likeability of certain cards introduces an interesting probabilistic perspective—people are more likely to choose well liked cards in a presented set, but this choice is not guaranteed. However, having those Liked cards in otherwise standard tuples should bias the likelihood of their selection, which can lead to a reduction in fishing questions needed. Therefore, the positioning of Liked cards throughout the cyclic deck becomes an additional constraint to optimize.

Finding and evaluating appropriate cyclic orderings is an extremely time consuming process for a human; a task arguably better handled by the search and optimization engine component of our framework. We chose Simulated Annealing (SA), a probabilistic search technique based on the metallurgical process of annealing, as the most appropriate technique available, as it has been shown to perform well in related search tasks such as the 8-Queens problem described in Russell and Norvig (2009). In computing, SA algorithms combine hill climbing and random walks to effectively traverse discrete search spaces in search of optimal solutions, and prove suitable for the discovery of cycles and Liked cards distributions. The categories that differentiate playing cards may be combined within a single deck, at different levels of the tree. Our approach allows for the flexible creation of decks to specification, allowing a performer to concentrate on designing an effective presentation, the importance of which is emphasized in Ortiz (1994).

The basic function of the SA procedure is to operate on a list of playing cards, swapping card positions to re-order the deck over many iterations, in order to maximize the longest consecutive sequence of cards that contains non-repeating sub-sequences of a specified length that uniquely identify themselves in the deck by the order of their categories (in the context of which level in the tree structure they are). A fifty two card cycle is the theoretical maximum for a fifty two card deck. As there may be more than one valid cycle for each set of categories selected, additional heuristics may be used to guide specific (not categorical) card placements, depending on the type of deck sought.

**Figure 10:**
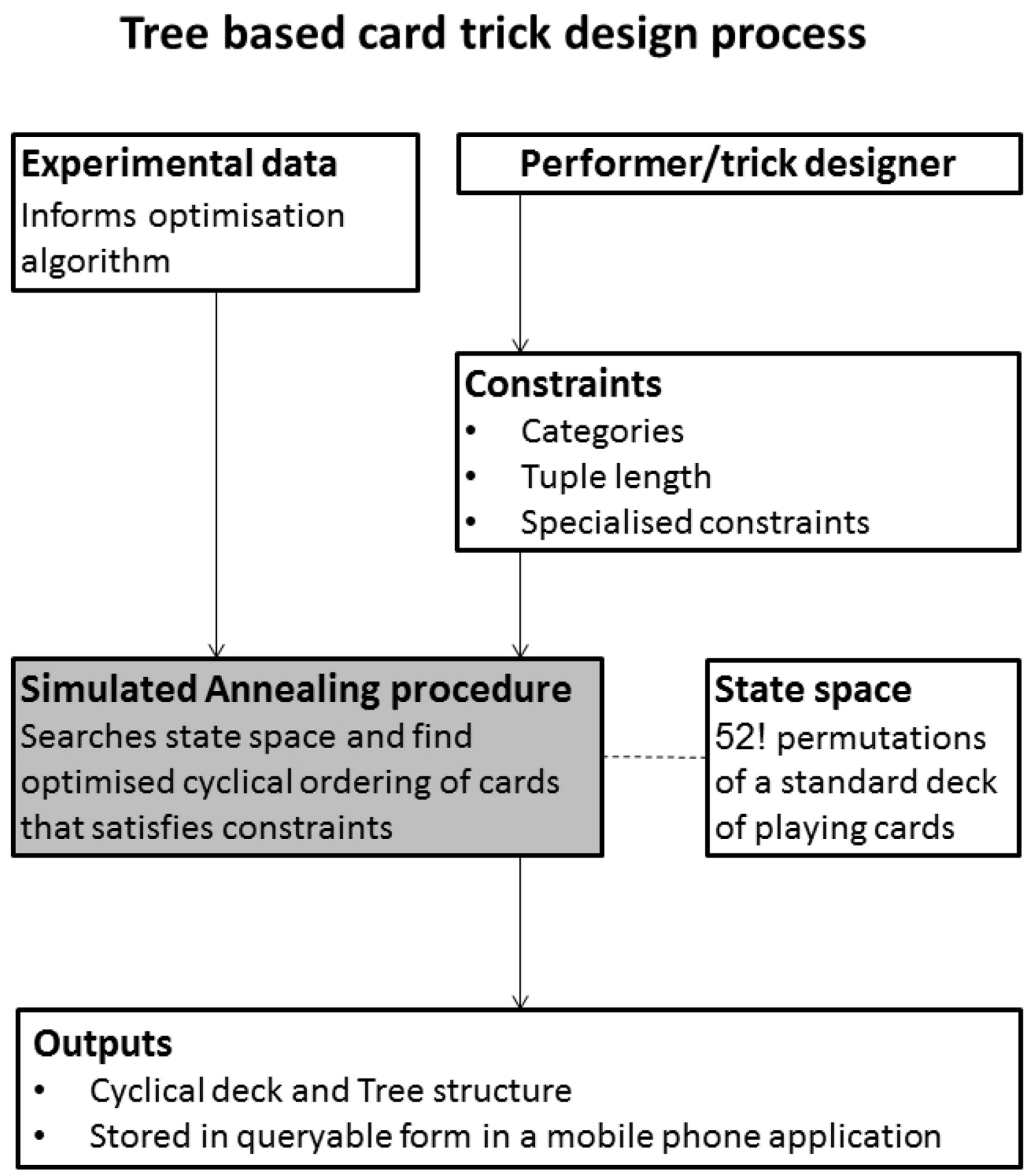
**The Simulated Annealing driven card trick design process**. Mathematical constraints about cyclical orderings of cards are combined with empirically derived psychological constraints about the likeability of certain playing cards by a SA algorithm that outputs an optimal deck according to operator specified psychological heuristics.

We employed our system, see Figure 10, to create and test a number of different decks, each with their own set of properties (categories, number of cards dispensed, etc).

**Figure 11:**
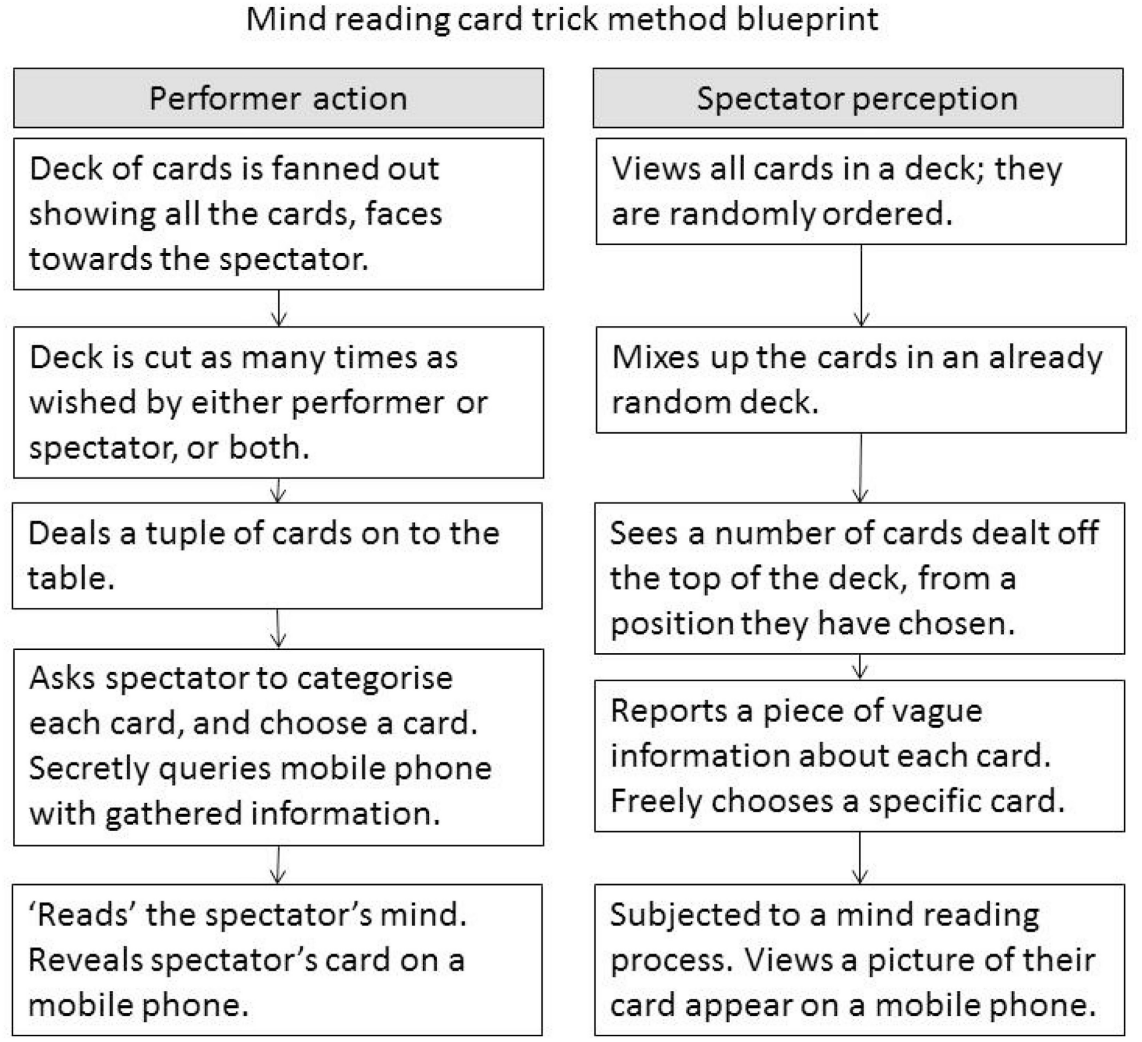
**This shows how the card trick can be performed, showing at each stage what the magician is doing, and what the audience is experiencing**.

Once a particular ordering of cards has been specified and found by the system, it must be deployed in performance (see Figure 11). Tricks featuring ordered decks of cards generally require memorization, and are usually limited by the mnemonic properties of the sequence. Cue cards as memory aids are common in commercial card tricks, for example the Simon-Eyes effect described in Aronson (1990). Human assistants or confederates can be deployed during such tricks, particularly if the method relies on some mathematical principle that requires information to be covertly available to the performer in some way; see Kleber and Vakil (2002), Simonson and Holm (2002) and Lee (1950b) for examples. The constraint on memorable orders can be lifted by using a digital assistant: in our case a mobile phone application that serves as both a cognitive aid for the performer of the type discussed in Dror and Harnad (2008); a queryable memory bank gimmick; and as a display to reveal the selected playing cards. The presence of the mobile phone in the trick could arouse suspicions in a spectator, specifically (and correctly) that the phone was being used as a queryable memory into which the results of the fishing questions were being fed to recover the card selection identity. To help disguise this process we implemented a faked passcode screen, which enables the magician to pass information to the app under the guise of unlocking the phone.

Further, we undertook experiments to gather data about which cards are most liked when presented in groups of four. Using this data as constraints in the SA search system, we also optimized a cyclical deck consisting of sequences of four cards arranged such that one and only one Liked card would appear in each tuple. During the trick, having identified the color of the four dealt cards, a spectator is asked to select their most liked card. Their card is revealed to them in the usual manner; however, it may take the performer up to four attempts to find the correct card, with an increasing probability of success at each stage but with clearly reducing magical impact. This principled probabilistic extension to the standard cyclic deck, which can reveal the selected card with minimal fishing but carries quantifiable risk, represents a novel element in the design of such tricks.

### 2.7. Card trick tree traversal results

To test the optimized decks produced by our system, we tasked it with finding a deck that could be used in an existing trick. We used Simon Aronson's Simon-Eyes effect, in Aronson (1990), for comparison. On average, in Aronson's trick, 4.04 questions will need to be asked before the magician knows the suit and value of the two dispensed cards. Using our SA procedure, a deck with a different set of categories has been found that, on average, will require 3.85 questions. Our deck will more frequently require one fewer question to arrive at the final two cards revealed by the magician. Both decks require a minimum of three questions, and a maximum of five. Aronson's ingenious deck was designed by him to be easily memorable, though Aronson does recommend the use of a cue card. In our effect we use a gimmicked prop, a mobile phone app, to traverse the tree structure.

### 2.8. The probabilistic deck; magic and probability

To test and optimize the various properties of our proposed probabilistic trick, based on the Liked category, we initially used the algorithm to construct a deck that had two categories, and a tuple length of four (i.e., four cards are dispensed). The categories used were Red, and Liked. Cards are described using the following key:

[*A*: *Ace*, *K*: *King*, *Q*: *Queen*, *J*: *Jack*, ♣: *Club*, ♢: *Diamond*, ♡: *Heart*, ♠: *Spade*]

Any four cards dealt from the deck will result in just one Liked card being dispensed. This should be the most likely card within that tuple for a spectator to pick (if carefully cued by the performer to select the card they like the most).

Olson et al. (2012) performed experiments showing people two cards at a time to determine the most liked card in each pair; we instead ran tests showing people four cards at a time, to match the setup of the trick; we ranked the cards based on our results, along with Olson's general results about most liked cards (Olson's conclusions are drawn from a much larger data set than ours, so we believe a combination of the results is a balanced approach to deriving something meaningful that can be used in a trick): “People like: Hearts, Spades, Aces, Face cards” Olson et al. (2012).

The 13 cards that made up our Liked category were, in rank order:

A♡,A♠,K♡,Q♡,J♡,K♠,10♡,Q♠,J♠,A♢,K♢,A♣,K♣

We configured the optimization engine heuristic rule set to maximize the likelihood of a spectator selecting the predicted liked card in a given tuple of cards. See Table 2.

**Table 2:** **Heuristics specified for the SA procedure, designed to maximize the likelihood of a spectator selecting the predicted liked card in a given tuple of cards**.

**Heuristic**	**Purpose**
Maximize the distance between the rank in the Likeability index of the Liked card in the tuple, and the highest rank from the other cards	High cards are strongly Liked. Two high cards in a set would make it less likely that one or the other would be selected as a Liked card. The predicted Liked card should be the highest ranking card in the set. The next highest ranking card should be as lowly ranked as possible.
Minimize the number of hearts in any one tuple	Hearts are strongly Liked. A predicted Liked card may not always be a Heart. Minimizing the number of Hearts in a tuple makes clashes with predicted Liked cards that are not Hearts less likely.
For Liked Clubs (i.e., the Ace of Clubs and the King of Clubs) minimize the number of red cards in the same tuple	Red cards are more likely to be Liked than black cards. To maximize the chances of a predicted Liked Club being selected by a spectator, there should ideally only be other black cards in the tuple.

The search process found the following optimized deck:

$$\begin{array}{l} 3♡,Q♡,J♣,2♠,6♡,A♣,4♣,5♠,7♣,10♡,3♠,2♣,9♣,K♠,4♠,\\ 6♢,Q♣,K♡,10♢,5♢,8♣,Q♠,2♡,3♢,5♣,A♢,8♠,J♢,10♣,\\ K♣,6♠,3♣,2♢,J♡,7♡,4♡,8♢,A♡,8♡,10♠,9♡,A♠,Q♢,7♠,\\ 4♢,K♢,6♣,7♢,9♢,J♠,9♠,5♡ \end{array}$$

We performed an online experiment with this deck sequence (*N* = 69), asking participants to select their most liked card in each tuple of four from the fifty two tuples in the cyclical deck. The participant group featured 23 males and 46 females. 35 respondents were from America, 26 from the UK, 2 from Canada, and 1 each from Australia, China, Finland, Libya, Lithuania, and Poland. Ages were approximately evenly distributed from 18 to 72, with a disproportionate number reporting 18 as their age (also the minimum age required for participation in the study). There was a good match between the predicted Liked card in any given tuple and the actual most liked card. The most liked card did not match the predicted most liked card for only one tuple: Eight of spades, Jack of diamonds (actual most liked card), Ten of clubs, King of clubs (predicted most liked). There is no obvious explanation for this, though the most likely is that the Jack of Diamonds is a relatively high ranking card appearing in the middle of the four cards, while the King of Clubs, in this tuple, appears at the edge.

### 2.9. The probability deck and invisible technology

We evaluated the magical impact of the probability deck and the feasibility of using a mobile phone gimmick for this trick by performing an experiment at a public event; the trick was performed for random spectators at a science festival (*N* = 116).

The average (mean) rating given to the trick was 3.28 (out of 4). The average (mean) rating given to participant's general view of magic was 3.53. The calibrated average (mean) was 3.04. It is interesting to note that this trick scored higher than both the magic jigsaw and the classic tricks discussed earlier. However, the participant's general rating of magic was also higher. This can possibly be attributed to the fact that the card trick was performed in a live setting, rather than in an online experiment, and that people choosing to sit down to see a trick were more likely to enjoy magic. The online participants may have been a more varied group (in terms of enjoying magic). The difference between the general rating and the card trick rating is 0.25 (this is similar to the jigsaw's difference rating of 0.24).

The words chosen by the participants, from our distilled list, to describe the card trick were overwhelmingly favorable. Participants were asked to circle at least one word from the list; some circled more. Of 164 words reported, 36 were “Surprised,” 47 “Amazed,” and 61 “Impressed.”

The free writing component of the evaluation allows participants to describe how the trick works, and to report any suspicious moments during performance. No participants were able to fully describe the operation of the trick. Around 10% guessed that the method relied on the ratio of red and black cards on the table. During the performance of the trick, the magician passes the information gleaned from the spectator (about the color of the cards dispensed from the deck, and their most liked card) to the app using a faked passcode screen into which a sequence of numbers representing the information is passed. Perhaps surprisingly, no participants mentioned the faked passcode screen as a possible medium of interaction between magician and phone.

During this probabilistic version of the trick it is inevitable that sometimes the wrong card will appear on the phone initially; it may take up to four attempts to reveal the correct card. Surprisingly, this had little effect on the enjoyment rating of the trick, though on the odd occasion that the full four attempts were taken, there was a reduction in the rating of enjoyment score reported. Otherwise, it is relatively easy for the performer to explain away the failures. For example, the magician might explain away a failure by saying that very advanced mind reading technology is being used, therefore naturally sometimes there are errors, and that they should try again, but this time the spectator must make a more concerted effort to visualize their card in their mind.

The mobile phone app we created that enables the presentation of the trick using various different decks with differing properties was successfully sold to magicians via a reputable magic shop in London, UK, at a price comparable to other apps. The app has recently been released on the Google Play store, and at the time of writing has sold a small number of copies, without yet being widely publicized. Two reviews have been posted, both awarding five stars out of five, along with a review comment from a magician: “Absolutely Brilliant.”

## 3. Discussion

We have introduced a general framework approach to designing and evaluating new magic tricks. The framework describes a method to integrate empirical data about human perception and cognition with artificial intelligence algorithms to create effects previously challenging for a human trick designer to produce, and allowing the inclusion of appropriate probabilistic techniques to enhance impact. The framework also provides a practical, principled way to objectively evaluate the output of the creation process. We note the success with which the tricks were accepted for inclusion in the inventory and sold to magicians in a reputable London magic shop. A copy of the jigsaw product is also archived in the library of the Magic Circle in London. We have shown two case studies that adapted the framework to specific types of trick, and successfully produced novel effects that were proven to be effective in real life scenarios. We believe this general approach to trick design is highly flexible and applicable to many different types of trick. There are many obvious avenues of further investigation, notably stage magic where the perpetual effects of shading or unusual body position may be included, large scale tricks on social media platforms, and close up magic that relies on particular attributes of the human visual system, for example through the modeling of misdirection or sensory illusions. There would appear to be a body of future research that could be fruitfully pursued investigating the human brain's apparent expectations of events, and coupling these observations with recent advances in probabilistic graphical methods in computer science, for example Bayesian Networks, to both produce tricks but also to test our understanding of the psychological processes. Applying these types of methods to the card trick presented here, or similar, could lead to new ways to create effective magic, and explore the cognitive mechanisms underpinning the spectator's experience. We have also shown that effects with significant magical impact can be implemented on computing devices; it might be expected that sophisticated technology would be incapable of producing a magical effect, as any seemingly impossible events could be easily attributable to the computer. Our investigations with the mobile phone card trick have shown that this is not necessarily the case; on the contrary, a new and wide range of possible effects intertwining the real and the virtual may be available to the modern magician with the right tools.

## Funding

We thank the Engineering and Physical Sciences Research Council (EPSRC). Ethics approval: QMREC2011/94 - Perception of computerized psychophysical stimuli.

### Conflict of interest statement

The authors declare that the research was conducted in the absence of any commercial or financial relationships that could be construed as a potential conflict of interest.
